# Development of a Preclinical Orthotopic Xenograft Model of Ewing Sarcoma and Other Human Malignant Bone Disease Using Advanced *In Vivo* Imaging

**DOI:** 10.1371/journal.pone.0085128

**Published:** 2014-01-07

**Authors:** Britta Vormoor, Henrike K. Knizia, Michael A. Batey, Gilberto S. Almeida, Ian Wilson, Petra Dildey, Abhishek Sharma, Helen Blair, I. Geoff Hide, Olaf Heidenreich, Josef Vormoor, Ross J. Maxwell, Chris M. Bacon

**Affiliations:** 1 Newcastle Cancer Centre at the Northern Institute for Cancer Research, Newcastle University, Newcastle upon Tyne, United Kingdom; 2 Newcastle upon Tyne Hospitals NHS Foundation Trust, Newcastle upon Tyne, United Kingdom; 3 Great North Children’s Hospital, Newcastle upon Tyne Hospitals NHS Foundation Trust, Newcastle upon Tyne, United Kingdom; University of Maryland, United States of America

## Abstract

Ewing sarcoma and osteosarcoma represent the two most common primary bone tumours in childhood and adolescence, with bone metastases being the most adverse prognostic factor. In prostate cancer, osseous metastasis poses a major clinical challenge. We developed a preclinical orthotopic model of Ewing sarcoma, reflecting the biology of the tumour-bone interactions in human disease and allowing *in vivo* monitoring of disease progression, and compared this with models of osteosarcoma and prostate carcinoma. Human tumour cell lines were transplanted into non-obese diabetic/severe combined immunodeficient (NSG) and Rag2^−/−/^γc^−/−^ mice by intrafemoral injection. For Ewing sarcoma, minimal cell numbers (1000–5000) injected in small volumes were able to induce orthotopic tumour growth. Tumour progression was studied using positron emission tomography, computed tomography, magnetic resonance imaging and bioluminescent imaging. Tumours and their interactions with bones were examined by histology. Each tumour induced bone destruction and outgrowth of extramedullary tumour masses, together with characteristic changes in bone that were well visualised by computed tomography, which correlated with post-mortem histology. Ewing sarcoma and, to a lesser extent, osteosarcoma cells induced prominent reactive new bone formation. Osteosarcoma cells produced osteoid and mineralised “malignant” bone within the tumour mass itself. Injection of prostate carcinoma cells led to osteoclast-driven osteolytic lesions. Bioluminescent imaging of Ewing sarcoma xenografts allowed easy and rapid monitoring of tumour growth and detection of tumour dissemination to lungs, liver and bone. Magnetic resonance imaging proved useful for monitoring soft tissue tumour growth and volume. Positron emission tomography proved to be of limited use in this model. Overall, we have developed an orthotopic *in vivo* model for Ewing sarcoma and other primary and secondary human bone malignancies, which resemble the human disease. We have shown the utility of small animal bioimaging for tracking disease progression, making this model a useful assay for preclinical drug testing.

## Introduction

The complex three dimensional anatomy of bone undergoes constant remodelling dependent upon the coordinated activities of multiple resident cell types. The growth of primary or metastatic cancer cells within the bone disturbs this equilibrium, producing clinically important changes in bone structure including aberrant new bone formation and bone destruction [1]. These changes may have significant clinical consequences such as severe bone pain, nerve compression syndromes, hypercalcaemia, cytopenias and pathological fractures, which may not only reduce quality of life but in many cases correlate with reduced survival [2], [3], [4]. Moreover, interactions with resident bone cells are critical for the intraosseous growth of the tumour.

Specific, and distinct, interactions with bone are central to the pathogenesis of Ewing sarcoma and osteosarcoma, the two most common primary bone sarcomas of children and young people. The majority of Ewing sarcomas arise in bone, with the femur, pelvis and humerus most often affected. Osteosarcomas are often localized to the metaphyseal region of long bones, with the region around the knee involved in around 60% of cases [5]. For both tumours, metastatic osseous spread is a feature of poor prognosis disease. While two thirds of Ewing sarcoma patients with localized disease can be cured, the 5-year event free survival in patients presenting with osseous metastases/bone marrow infiltration is only 10–20% [6]–[8]. Furthermore, bone tissue is one of the most common sites of metastatic disease in common cancers such as carcinoma of the prostate. More than two thirds of patients with advanced prostate carcinoma develop bone metastases conferring a poor prognosis, with the axial skeleton most frequently affected [9].

The pathophysiology of bone remodelling and intraosseous tumour growth in bone cancers such as Ewing sarcoma is still unclear and requires further investigation. Similarly, while the basic mechanisms by which tumours such as prostate carcinoma home to the bone marrow cavity and interact with the cells in the metastatic niches of bone have been explored, a detailed understanding is still lacking [10]–[14]. This lack of understanding has prevented the development of effective treatments for osseous disease. The validation of clinically relevant preclinical models will provide tools with which to both study the mechanisms involved in disease progression, and examine novel treatments directly targeting the interaction of tumour cells with the bone microenvironment.

Currently, many preclinical models of bone cancer, particularly those of Ewing sarcoma, use subcutaneous or intramuscular xenografts, which clearly do not mirror the site of disease in patients [15]. Metastatic intravenous models of Ewing sarcoma in non-obese diabetic/severe combined immunodeficient (NSG) mice show a pattern of disease spread similar to that found in patients, providing a suitable system for studying metastasis of this disease. However, only 23% of the experimental mice in these studies developed assessable bone metastases [16]. To facilitate better preclinical study of primary bone tumours and their metastatic spread, and of bone metastases of cancers such as prostate carcinoma, it is desirable to develop orthotopic models that involve direct injection of cancer cells at the clinically relevant site. Intrafemoral injection in immunocompromised mice provides a technically feasible and reproducible approach to such models [17] giving rise to tumours that are detectable by palpation or *in vivo* imaging of the animals and closely resemble those found clinically. Intrafemoral injection is a minimally invasive and non-traumatic procedure, as are some intratibial transplantation techniques [18]; [19]; [20].

The major difficulty in using preclinical orthotopic models of any cancer has been the measurement of disease burden in a non-accessible site. The use of *in vivo* imaging offers the opportunity to detect and monitor development and progression of the disease. Small animal imaging technologies such as positron emission tomography (PET), computed tomography (CT), magnetic resonance imaging (MRI) and bioluminescent imaging are thus becoming powerful tools to monitor tumours in orthotopic disease models. In this study we have therefore used intrafemoral injection, combined with *in vivo* imaging approaches, to develop a preclinical orthotopic model for Ewing sarcoma. We have compared the bone changes induced by Ewing sarcoma to those induced by osteosarcoma and prostate carcinoma to validate the model as one in which specific interactions of both primary and secondary bone tumours with the bone itself can meaningfully be investigated.

## Materials and Methods

### Definition of Experimental Outcomes

Primary outcomes assessed in this study were: tumour formation and growth, destructive bone changes mirroring the human disease (histology, CT findings) and imaging of primary tumour. The secondary outcome assessed was the detection of distant tumour spread by imaging techniques.

### Cell Lines and Cell Culture

Two established human Ewing sarcoma cell lines were used for transplantation of mice. TC-71 originated from a local Ewing sarcoma relapse in the humerus [21]. VH-64 cells were established from a malignant pleural effusion in a patient with Ewing sarcoma of the metatarsal bone [22]. SaOS-2 is an established osteosarcoma cell line derived from a primary osteosarcoma [23]. PC3M is an established prostate carcinoma cell line derived from a mouse liver metastasis arising from xenotransplantation of an osteolytic bone metastasis of a prostatic adenocarcinoma [24], [25].

All cell lines were cultured in Roswell Park Memorial Institute (RPMI) 1640 medium (Sigma, Germany) containing 10% FCS (foetal calf serum) and 2 mM glutamine at 37°C in a humidified atmosphere with 5% CO_2_. Ewing sarcoma cells were grown in collagen-coated tissue culture flasks.

### Transduction of TC-71 Cells

A protocol described previously [26] to transduce lymphoblastic leukaemic blasts was adapted to transduce Ewing-sarcoma cells with a lentiviral vector (pSLIEW) encoding both enhanced green fluorescent protein (EGFP) and firefly Luciferase (fLuc), allowing *in vitro* analysis and cell sorting (EGFP) and *in vivo* bioluminescent imaging (fLuc). The SLIEW virus was produced by transfection of HEK293T cells with equimolar amounts of a packaging vector (pCMVΔ8.91), an envelope vector pMD2.G and the pSLIEW transfer vector. For transductions, TC-71 cells were grown for 24 hours on collagen coated 48-well plates before 100 µl viral suspension and 8 µg/ml polybrene were added and viral transduction was performed by spinfection at 1500 g for 2 h at 32°C. Plates were further incubated at 37°C/5%CO_2_ and medium was replaced by fresh medium the day after. Transduction efficiency (percentage of EGFP positive cells) was assessed 3–4 days after transduction and prior to i.f. injection by flow cytometry (FACSCalibur, Becton Dickinson, Oxford, UK) using Cell Quest Pro Software, version 5.2.1. Either 25% EGFP positive or sorted 100% positive EGFP cells were used in 3 experiments.

### Ethics Statement

All animal studies were performed by personnel who had completed approved Home Office training and held current Personal Licences under the Animals (Scientific Procedures) Act 1986. All *in vivo* experiments were performed in line with current Home Office regulations and compliant with the 3R principles (Home Office license number PPL 60/3846) and therefore no further ethics approval was required. Animals were kept under specific pathogen free conditions, and all experimental manipulations with mice were performed under sterile conditions in a laminar flow hood except imaging. Intrafemoral injections and all imaging procedures were performed on anaesthetized mice and all efforts were made to minimise suffering.

### Mice and Intrafemoral Transplantation

NSG mice were transplanted with 1×10^6^ cells at a median age of 12 weeks (range 10–3 weeks), Rag2^−/−^ γc^−/−^ mice with 1×10^3^–1×10^6^ cells at 12 to 17 weeks. For intrafemoral transplantation, cells were injected directly into the right femur of male NSG mice (Jax® mice strain name: NOD.Cg*-Prkdc^scid^ Il2rg^tm1wjl^*/SzJ) or Rag2^−/−^ γc^−/−.^mice (Jax® mice strain name: C;129S4-*Rag2^tm1.1Flv^ Il2rg^tm1.1Flv^*/J). Both mouse strains lack NK cell, B and T lymphocyte activity [27]; [28], minimising the chance of xenograft rejection. Rag2^−/−^ γc^−/−^ mice were chosen in addition to NSG mice as the latter harbour a DNA-PK defect which could confound future experiments using DNA repair inhibitors. The procedure for intrafemoral injection was previously described [17]; [29].

Briefly, mice were anaesthetized with an Isofluorane-oxygen gas mixture (approx. 2.5% Isofluorane and 0.5l O_2_ per minute). Carprofen (Rimadyl®, Pfizer, Surrey, UK), 50 µg per 10 g body weight, was injected subcutaneously as an analgesic. The right knee was flexed, the femur was punctured with a 25 Gauge needle and cells were injected with a 30 Gauge insulin syringe through the knee joint into the right femur. Control transplantations were performed by injection of the right femur with medium only. For experiments using NSG mice, 1×10^6^ cells were injected in a 30 µl volume. For experiments using Rag2^−/−^ γc^−/−^ mice, either 1×10^4^–1×10^6^ cells in 30 µl medium (experiments 1 and 2) or 1×10^3^–1×10^4^ cells in 10 µl medium (experiment 3) were injected.

### Histology

Organs and tumour masses were fixed in 10% neutral buffered formalin (4% formaldehyde in phosphate buffered saline). Fixed hind limbs bones were decalcified *in situ* in 10% formic acid or in 14% neutral EDTA solution until suitable for microtomy. Subsequently, tissues were embedded in paraffin, and 4–5 µm thick sections were prepared and stained with hematoxylin and eosin.

### CT Imaging

After i.f. injection and before reaching protocol limits, each mouse was subjected to two or up to a maximum of five CT scans using the Bioscan NanoCT scanner (Bioscan, Paris, France). These were performed before each PET scan using a transferable animal bed so that animals were kept in the same position to allow accurate subsequent fusion of PET and CT image datasets.

The field of view for the scans was 150 mm×75 mm. Parameters for whole body images were: standard resolution, 180 projections, 55 kVp, 1000 ms exposure time and 10–12 min acquisition time, leading to a dose of ∼150 mGy. The parameters for scans of a higher resolution taken from just the lower limbs were: ultra-fine resolution, 360 projections, 45 kVp, 400 ms exposure time, 2–3.5 min acquisition time, leading to a radiation dose of ∼105 mGy. The images were reconstructed using Exact Cone Beam FBP (Filtered Back Projection) with the SheppLogan filter. The resulting voxel/pixel size is 0.10/0.096 mm.

### PET Imaging

PET imaging was performed on 5 mice injected with Ewing sarcoma cells, on 5 mice injected with osteosarcoma cells, on 2 animals injected with prostate cancer cells and on 2 control animals. For 5 mice, the first PET scan was performed approximately three weeks after intrafemoral injection of tumour cells with follow-up PET scans 3–4 weeks after the initial PET, depending on the development of disease. All other animals had PET scans performed around 7 weeks after i.f. injection. 2-[^18^F]fluoro-2-deoxy-D-glucose (FDG) was purchased from IBA Molecular UK Ltd. (Sheffield, UK). The tracer was diluted with sterile saline if required. PET scans were performed with a high-resolution Philips Mosaic HP preclinical PET system (Philips Medical Systems, Eindhoven, The Netherlands) with a field of view of 128×120 mm. Before tracer injection, mice were anaesthetised either with inhalation of Isoflurane-oxygen gas mixture (approx. 2% Isoflurane, 0.5 l/min oxygen) or intraperintoneal injection of ketamine (50–75 mg/kg) plus medetomidine (0.5–1.0 mg/kg) in a volume of 10 ml/kg. Mice anaesthetized with Isofluran/oxygen were kept under anaesthesia for the entire experimental procedure using a Minerve Animal Anaesthesia System (Equipement Vétérinaire Minerve, Esternay, France). Rectal temperature was maintained at physiological values by use of a heating pad between the start of the anaesthesia and the experimental procedures. Scans were performed 1 hour after an intravenous injection of 10 MBq FDG. Within the machine, the body temperature was maintained by heated air flow through the animal bed. All mice were observed until fully recovered from anaesthesia.

Reconstruction of PET images was performed using the 3D-RAMLA algorithm with no attenuation correction. Quantitative PET imaging results are reported in terms of standardised uptake value (SUV) with both SUVmean (mean of pixel SUV values within a defined region of interest) and SUVmax (maximum pixel value in that region of interest) presented.

### MR Imaging

One week after the last CT scan (8–9 weeks), 2 mice transplanted with Ewing sarcoma (VH-64) and 2 injected with prostate carcinoma (PC3M) cells underwent a final MRI scan. Two mice transplanted with osteosarcoma cells (SaOS-2) and 1 control mouse were scanned after 12 weeks. MRI scans were performed with a high-resolution 7Tesla Varian MR system (Varian Inc., Palo Alto, USA) using a Rapid 72 mm quadrature 1 H volume coil (RAPID Biomedical GmbH, Rimpar, Germany). Mice were anaesthetised for the entire experimental procedure by Isoflurane/oxygen inhalation (approx. 2% Isoflurane, 0.5 l/min oxygen).

Parameters for the lung scans were: fast spin echo sequence with respiratory gating, repetition time (*T_R_*) 2000–2500 ms, effective echo time (*T_E_*) 37–48 ms, matrix 256×256 with echo train length 8, field of view 40×40 mm, in plane resolution 0.16 mm and slice thickness 2 mm. Parameters for the leg scans were: spin echo sequence, *T_R_* 500 ms, *T_E_* 17 ms, field of view 30×35 mm, in plane resolution 0.13 mm and slice thickness 2 mm, giving a T1 weighted sequence.

For calculation of tumour volumes the software Vnmr J (Varian/Agilent) Version 3.1A was used. 1 H MR images (fast-spin echo with TR = 2000 ms, effective TE 39–48 ms, echo train length 8) were obtained from 1 mm or 2 mm thick slices with a field of view 40 mm×40 mm and matrix size 256×256, corresponding to an in-plane spatial resolution of 0.16 mm. For each slice in which tumour was identified, a region of interest was drawn around the tumour and the area of that region determined using the region of interest tool in VnmrJ. The areas of all tumour-containing slices were summed to estimate the tumour volume.

### Bioluminescent Imaging

Rag2^−/−^ γc^−/−^ mice transplanted with luciferase-expressing TC-71 cells underwent weekly non-invasive whole-body imaging starting 1 week after intrafemoral injection using the IVIS Spectrum Imaging system (Caliper Life Sciences, Hopkington, MA, USA). Mice were injected i.p. with 3 mg/100 µl of luciferin solution before being anaesthetized for the procedure. To avoid unintentional injection of the substrate into the gut, mice in experiment 3 were injected subcutaneously (s.c.) with 150 µg luciferin/g body weight. Ten or twelve minutes after i.p. or s.c. injection, images were acquired for 0.5 to 300 seconds. The photon emission from the animals was captured and quantitated in p/s/cm^2^/sr using *Living Image* software (Version 4.3.1., Caliper LifeSciences).

## Results

### Success of Transplantation Strategy and Time Course of Induced Tumours

Each cell line injected gave rise to tumour within the medullary cavity of the femur, bone destruction, and growth of extra-medullary tumour masses showing variable degrees of necrosis. The tumour growth of transplanted prostate carcinoma cell line PC3M required euthanasia of experimental mice 29 days (median) after transplantation, the shortest time to reach protocol limits, whereas animals transplanted with SaOS-2 osteosarcoma cells lived 70 days (median) after transplantation (Table 1). NSG mice transplanted with Ewing sarcoma cell lines survived 31–38 days (median), Rag2^−/−^ γc^−/−^ mice survived for 43.5 days (median). With the exception of one Rag2^−/−^ γc^−/−^ mouse and the control mice transplanted with medium alone, all experimental animals developed tumours at the site of injection (Table. 1). Taken together, both mouse strains proved to be excellent recipients for bone cancer orthotopic xenografts with a “take rate” of almost 100%.

**Table 1:** Experimental mice for the development of an orthotopic model of bone malignancies.

Transplanted cell line	Numberof mice	Age at transplantation,weeks: median, (range)	Time to reach protocol limits, days: median, (range)	Tumour formation,%	Dominant histological feature
**VH-64**	7	12 (11–12)	38 (31–55)	100% (7/7)	Extensive spicules of reactive new bone
**TC-71**	6	12 (11–12)	31 (15–42)	100% (6/6)	Reactive new bone and cortical destruction
**TC-71 in Rag2^−/−^** **γc^−/−^ mice**	13	13 (12–17)	43.5 (4–62)	92% (12/13)	As for TC-71
**SaOS-2**	5	12 (10–12)	70 (52–85)	100% (5/5)	Malignant new bone formation
**PC3M**	7	12 (12–13)	29 (29–55)	100% (7/7)	Osteoclastic cortical lysis and reactive new bone
**Medium**	5	11 (11–12)	N.A. (sacrificed>day 85)	0% (0/5)	Normal findings

VH-64 and TC-71: Ewing sarcoma cell lines; SaOS-2: osteosarcoma cell line; PC3M: prostatic adenocarcinoma cell line; Medium: mice were injected with medium alone. N.A.: not applicable. All mice were NSG mice unless stated otherwise.

### Histology and Corresponding CT Findings

Each different type of cell line transplanted had characteristic predominant effects on the bone, reflecting the range of changes seen in patients with these tumours. Supporting Fig. S1 illustrates the CT and histological appearances of control mice injected with medium alone for comparison. The histological and CT imaging appearances of these control mice were normal.

#### VH-64 Ewing sarcoma cell line

**Figure 1:**
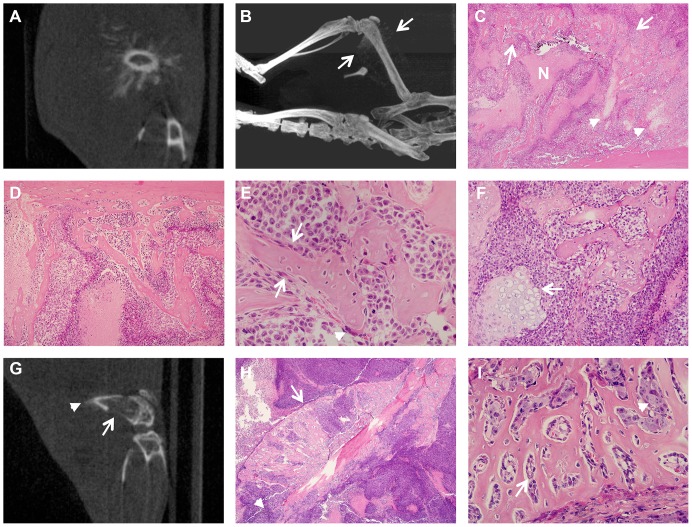
Prominent reactive bone formation following transplantation of Ewing sarcoma cell lines VH-64 (A–E) and TC-71 (F–I). **A**, Representative CT image of a VH-64 transplanted femur 42 days after transplantation showing a “sunburst” pattern of reactive bone formation. **B**, Representative Maximum Intensity Projection CT image of a mouse with a VH-64 tumour 52 days after transplantation showing a “sunburst” pattern of reactive bone formation on the transplanted femur (between arrows, and extending proximally along the femur). **C**, Extensive reactive bone (arrows) arcing through a VH-64 tumour accompanied by foci of cartilage (arrowheads). Tumour is partly necrotic (N) (H&E, original magnification 20×). **D**, Reactive bone emanating from femoral cortex (top) within a VH-64 tumour (H&E, original magnification 100×). **E**, Reactive new bone formation partially lined by osteoblasts (arrows) amongst VH-64 tumour cells. A single multinucleate osteoclast (arrowhead) is also present (H&E, original magnification 400×). **F**, Reactive bone and a focus of cartilage (arrow) amongst VH-64 tumour cells (H&E, original magnification 200×) **G**, Representative CT image of a TC-71 transplanted femur 15 days after transplantation showing cortical destruction (arrow) and possible reactive new bone formation (arrowhead). **H**, Marked reactive new bone formation within a TC-71 tumour, beneath the raised periosteum (arrow). Cortical destruction is seen distally (arrowhead) (H&E, original magnification 40×). **I**, Reactive new bone in a TC-71 transplanted mouse, lined by osteoblasts (arrow) clearly distinct from infiltrating tumour cells (arrowhead) (H&E, original magnification 400×).

CT images of the femurs injected with VH-64 cells were notable for the presence of new calcified material extending outward from the surface of the injected femur (Fig. 1A, B). In most cases these manifested as prominent spicules radiating perpendicularly from the surface in a “sunburst” or “sunray spicule” pattern. This pattern tended to overshadow destructive changes on CT scans. Histological examination (Fig. 1C–F) showed numerous and extensive spicules of reactive bone amongst tumour cells, but often associated with osteoblasts, extending in a perpendicular manner away from, and continuous with the periosteal surface of the femur. In many areas, these were associated with cortical destruction and appeared beneath a raised periosteum, but such bone was sometimes present amongst tumour cells significantly away from the femur. This reaction was also associated with the formation of islands of reactive cellular cartilage, often within bands of loose fibrous tissue, amongst the tumour mass. In some mice similar, but less marked, changes were also seen on the non-transplanted fibula.

#### TC-71 Ewing sarcoma cell line

Histological examination of femurs injected with TC-71 cells showed similar, but generally less extensive, reactive new bone formation, typically sub-periosteal and in association with cortical destruction. Reactive cartilage was observed only occasionally and very focally. On CT scans, bone destruction was the dominant appearance although pericortical new bone was faintly evident in some mice (Fig. 1G–I).

#### SaOS-2 osteosarcoma cell line

**Figure 2:**
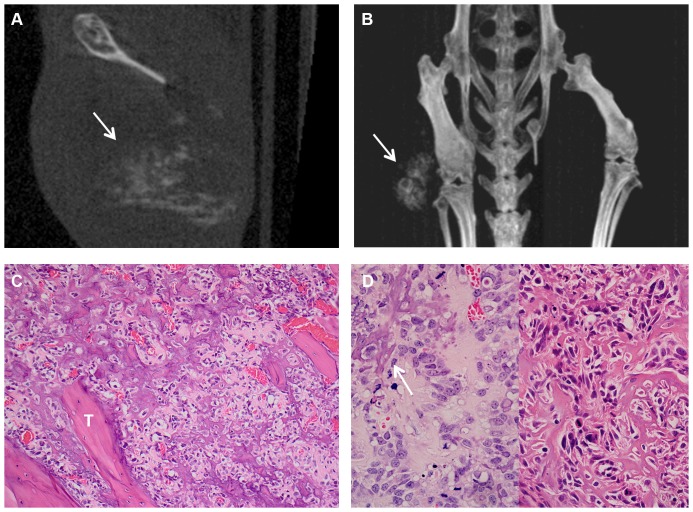
“Malignant” bone formation within the tumour mass after SaOS-2 transplantation. **A**, Representative CT image 70 days after intrafemoral transplantation of SaOS-2 cells showing “malignant” bone formation within the extraosseous tumour mass (arrow). **B**, Representative Maximum Intensity Projection CT image of a mouse bearing a SaOS tumour 70 days after transplantation, showing “malignant” bone formation within the extraosseous tumour mass (arrow). **C**, “Malignant” bone forming amongst tumour cells in the medullary cavity, showing appositional deposition (scaffolding) on existing bone trabeculae (“T”) (H&E, original magnification 200×). **D**, Left panel: malignant tumour cells amongst pale eosinophilic osteoid which is partially mineralised (arrow) (H&E, original magnification 400×). Right panel: fine, randomly arborising strands of mineralised osteoid (“malignant” bone) amongst SaOS-2 tumour cells (H&E, original magnification 400×).

In keeping with its cellular origin, the osteosarcoma cell line SaOS-2 was unique amongst the lines used in producing bone from the tumour cells themself (so called “malignant” bone). This manifested on CT scans as rather granular or fluffy calcification present within the extramedullary tumour mass, sometimes well away from, and obviously discontinuous with, the femoral surface (Fig. 2A, B). Histology showed large areas in which SaOS-2 tumour cells were separated and surrounded by pale eosinophilic unmineralised osteoid and mineralised “malignant” bone with a fine, arborising, spiculate pattern. In some areas, particularly within the medullary cavity, “malignant” bone showed appositional deposition on reactive or normal bone (scaffolding) (Fig. 2C, D). The bone changes seen histologically following intrafemoral growth of SaOS-2 cells also included focal sub-periosteal and intra-medullary reactive new bone formation, as well as regions of more diffuse cortical thickening. It was difficult radiologically to distinguish periosteal reactive new bone from the “malignant” bone formed by these tumours.

#### PC3M prostatic adenocarcinoma cell line

**Figure 3:**
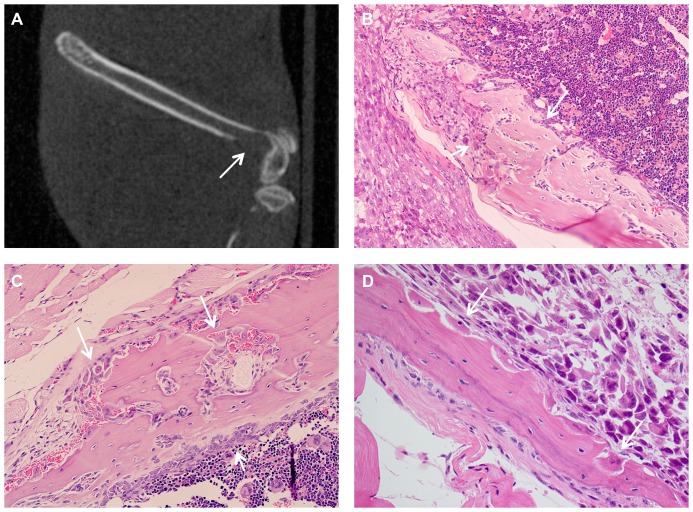
Osteolytic lesions caused by injection of PC3M. **A**, Representative CT image 29 days after transplantation of PC3M cells, showing lytic destruction of the femur (arrow) (sagittal section). **B**, Lytic destruction of the femoral cortex associated with numerous osteoclasts (short arrow) and focal reactive bone formation (long arrow) (H&E, original magnification 200×). **C**, Lysis and virtual destruction of the bone by numerous osteoclasts on the periosteal surface (long arrows), with osteoblastic reaction on the medullary surface (short arrow) (H&E, original magnification 200×). **D**, Lysis of bone by osteoclasts in resorption pits on the medullary surface (arrows), clearly distinct from intrafemoral tumour (H&E, original magnification 400×).

As a comparison to the primary bone tumour cell lines, the prostate carcinoma cell line PC3M was used to create a model for metastatic bone lesions. By CT scanning, injected femurs showed clear signs of resorption of bone leading to pathological fractures of the femur in 2 of 7 injected mice (Fig. 3A). Histological examination (Fig. 3B–D) of PC3M injected femurs often showed focal cortical destruction and exuberant extra-medullary tumour growth with a relatively low intra-medullary tumour burden. PC3M-induced osteolysis was characterised by numerous resorptive pits formed by active osteoclasts on the surface of the bone. These changes occurred on either the periosteal or endosteal cortical surfaces, adjacent to the tumour mass, but could be separated from the tumour by periosteum and skeletal muscle, suggesting that PC3M cells could induce osteoclast activation even at a distance from the bone. Osteoclastic bone resorption was often accompanied by a compensatory osteoblastic reaction and reactive new bone formation on the opposite aspect of the cortex. While other injected cell lines also induced bone destruction as large tumours developed, this was not characterised by the osteoclastic osteolysis induced by PC3M.

### PET Imaging of Experimental Mice

**Figure 4:**
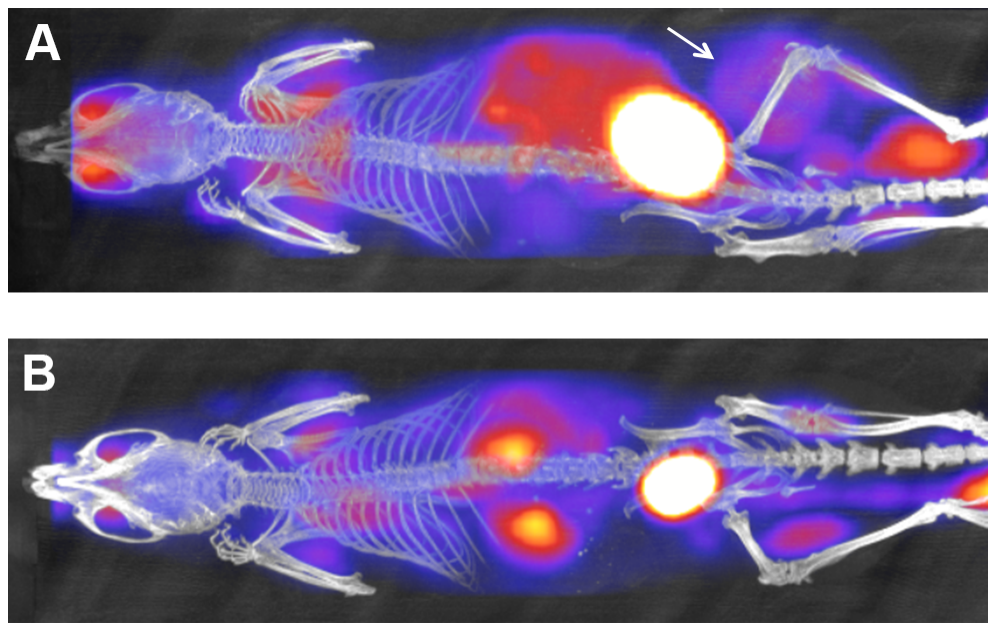
PET-CT imaging of primary tumour. **A**, PET/CT image of a mouse with a VH-64 tumour following intrafemoral transplantation. Image taken 52 days after transplantation, tumour marked with a white arrow (Maximum Intensity Projection CT image overlaid with FDG-PET image, dorsal view, ketamine/medetomodine anaeshesia). **B**, PET/CT image of a mouse injected with medium alone, also 52 days after i.f. injection, no tumour visible (Maximum Intensity Projection CT image overlaid with FDG-PET image, dorsal view, ketamine/medetomodine anaeshesia).

Whole body PET/CT imaging of 4 mice transplanted with VH-64 Ewing sarcoma cell was used to characterise primary tumours and detect possible distant tumour spread. In all cases, eyes, heart, intestines and in some animals the brain or eyes/lacrimal glands showed FDG accumulation. Bladder and kidneys showed FDG signal due to renal excretion of the isotope. Muscles showed background signal to a lesser extent in some cases. Fig. 4 shows a representative PET/CT image of a VH-64 tumour bearing mouse taken 52 days after transplantation (Fig. 4A) in comparison with a day 52 PET/CT image of a mouse injected with medium alone (Fig. 4B). PET/CT detected a tumour surrounding the right femur in all mice injected with VH-64 cells, but not in control mice. However, FDG-uptake was relatively low in the tumour (SUVmean = 1.5, SUVmax = 2.0 in the VH-64 tumour shown in Figure 4A) and it should be noted that FDG uptake in other parts of the body was substantially higher (SUVmean/SUVmax: bladder = 14.7/20.6; intense gut region = 2.4,/3.5; muscle 0.7,/1.4). In three VH-64 tumour bearing mice the mean (+/− sd) values of SUVmean were as follows: tumour 0.9+/−0.1; muscle 0.6+/−0.2; heart 13.7+/−2.9; bladder 14.0+/−9.8; gut 2.9+/−0.7). Therefore, tumour visualisation in the primary (intrafemoral) site was only feasible because of low uptake in leg muscle of anaesthetised mice. Mice injected with SaOS-2 or PC3M cells, and 1 mouse injected with TC-71 cells, did not show any evidence of increased FDG uptake in the region of tumour formation.

#### Choice of anaesthetic for FDG-PET imaging

**Figure 5:**
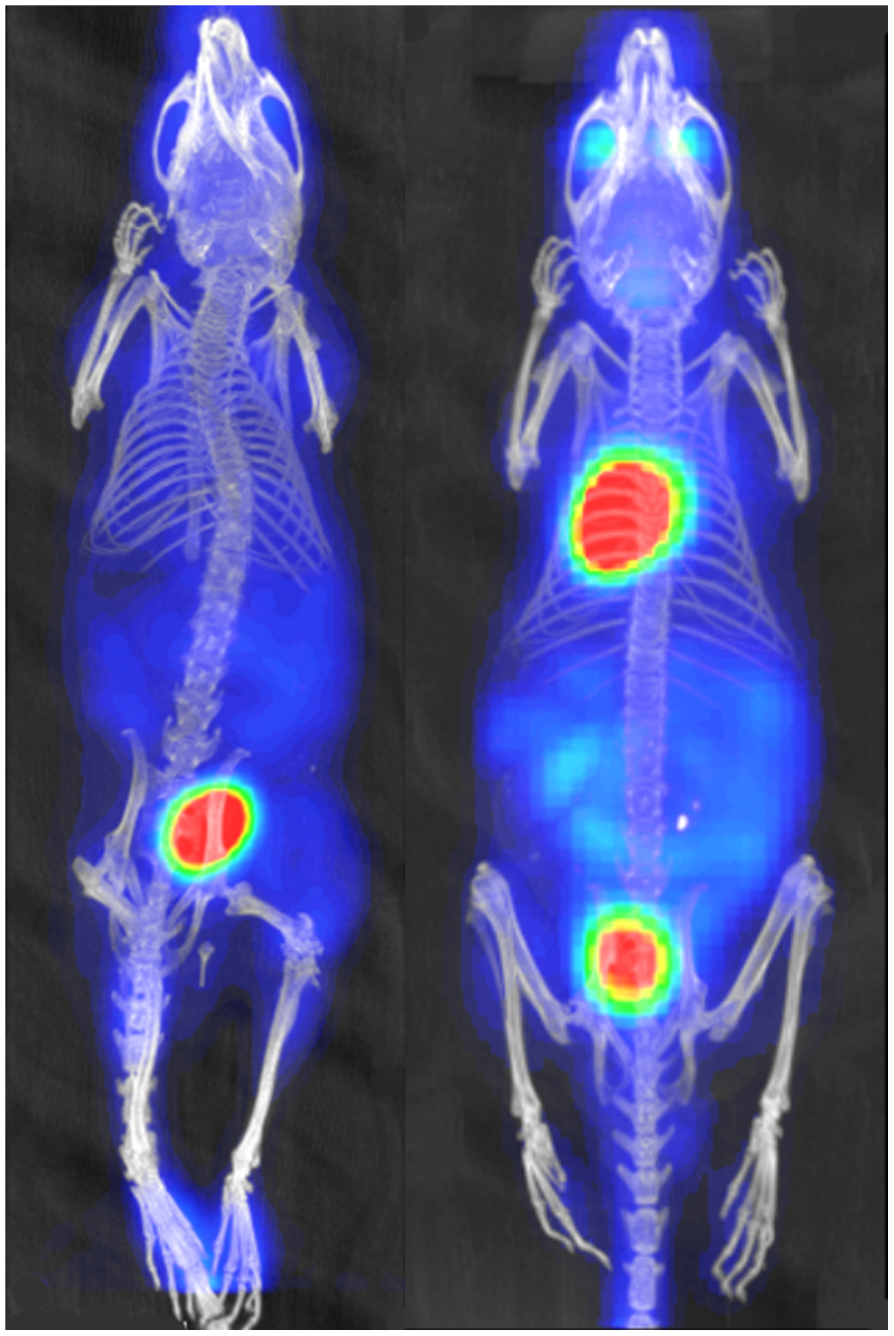
Comparison of different anaesthetic techniques. Representative FDG-PET images of mice anaesthetized with isofluorane (right mouse) versus ketamine/medetomidine (left mouse) showing a marked reduction in cardiac FDG uptake in the latter.

The high FDG uptake in the heart of mice studied anaesthetised with isofluorane made it impossible to judge whether there was pathological involvement of the lung. A substantial reduction in heart FDG uptake was achieved by changing from isoflurane anaesthesia to the use of ketamine/medetomidine, as shown in Figure 5. However, no lung deposits could be visualised by FDG-PET using either anaesthetic technique, even when these were histologically proven.

Overall, PET imaging was in principle able to detect primary bone tumours, but given the limitations described above this method was neither suitable for monitoring of tumour burden at the primary site, nor as a whole body “screening tool” for distant metastases.

### Documentation of Soft Tissue Involvement using MRI

**Figure 6:**
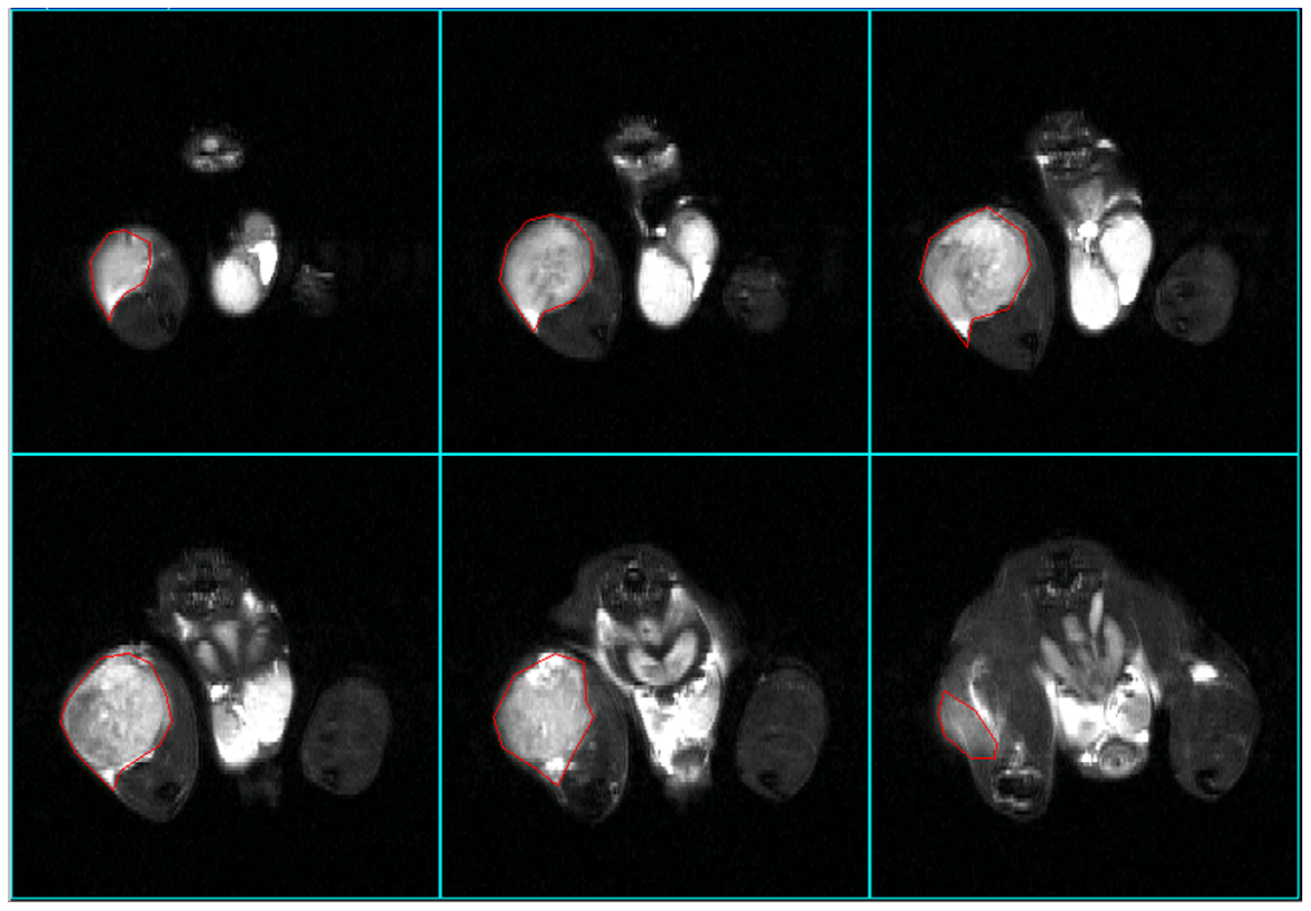
MR imaging of primary tumour with measurement of tumour volume. Measurement of a VH-64 Ewing sarcoma of the injected femur with a large extra-osseous tumour component. The regions of interest (within red markings) were measured in sequential 2 mm thick slices that showed tumour, and by adding the volumes of individual slices the total tumour volume was calculated. In the depicted case the estimated total volume was 492 mm^3^ (tumour in 6 slices; region of interest per slice: 22 mm^2^, 42 mm^2^, 57 mm^2^, 59 mm^2^, 52 mm^2^, 14 mm^2^; total area = 246 mm^2^; slice thickness = 2 mm; estimated total volume = 492 mm^3^).

Where possible tumour mass was assessed by MR imaging before dissection. In all cases, MRI was a useful technique to visualize the tumour mass growing at the site of injection (Fig. 6, VH-64 tumour; Supporting Fig. S2, SaOS-2 tumour and control mouse injected with medium alone and showing normal MRI appearances for comparison). It was possible to calculate tumour volumes by selecting the region of interest in sections that showed tumour mass and summing the volumes of tumour determined in individual slices (Fig. 6).

**Figure 7:**
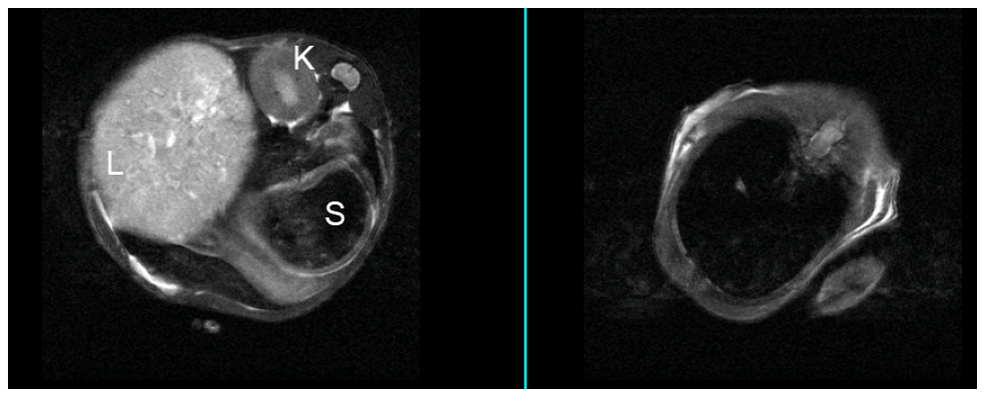
MR images of liver deposits and lungs using respiratory gating. MRI imaging performed 47 days after injection of a mouse with VH-64 cells, providing an example of respiratory gating to obtain good quality images. 2 out of 25 adjacent 2 mm slices (selected slices 12 mm apart) are presented showing upper abdomen (left panel), revealing a large liver tumour, and lungs (right panel). Despite slight movement artifacts on the thoracic images there is good visualisation of the lung fields. L: liver; K: kidney; S: stomach.

MRI also has a potential application for detection of pathology, including tumour metastases, in the lung and liver. In those regions it was necessary to obtain images using respiratory gating and, in general, good quality images could be obtained with minimal movement artifact. Figure 7 shows two out of 25 adjacent axial 2 mm slices, 12 mm apart, from a mouse with an intrafemoral VH-64 Ewing tumour. The left image is an axial upper abdominal slice depicting liver (which is largely replaced by a tumour mass), kidney and stomach, and the right image is a slice through the thorax showing the lungs. Although there are slight residual movement artifacts in the thoracic images, visualisation of any lung tumours >2 mm diameter could be expected. Thus these images are examples of both good quality respiratory gating and detection of distant sites of disease by MRI. Out of 6 tumour bearing mice that underwent an end-of life MRI scan, 3 (1 VH-64 mouse and 2 PC3M mice) had detectable liver or abdominal disease. In summary, MRI is an excellent method to obtain anatomical detail of primary tumours and their distant metastases, and the ability to measure tumour volumes allows for exact monitoring of disease progression.

### Bioluminescent Imaging

**Figure 8:**
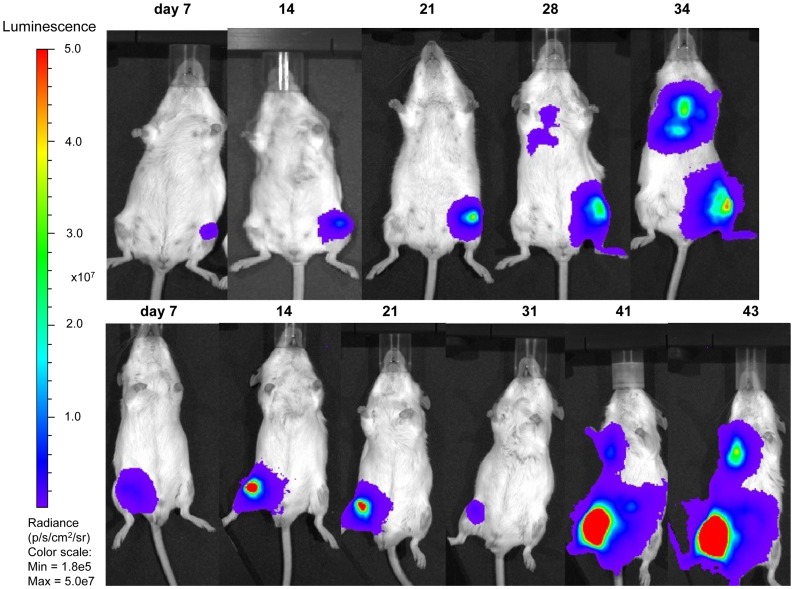
Bioluminescent imaging of Rag2^−/−^ γc^−/−^ mice intrafemorally transplanted with transduced TC-71 cells. Weekly bioluminescent imaging of two Rag2^−/−^ γc^−/−^ mice transplanted with 5×10^5^ transduced TC-71 cells in a volume of 30 µl (25% EGFP positive cells, top row and sorted 100% EGFP positive cells, bottom row).

**Figure 9:**
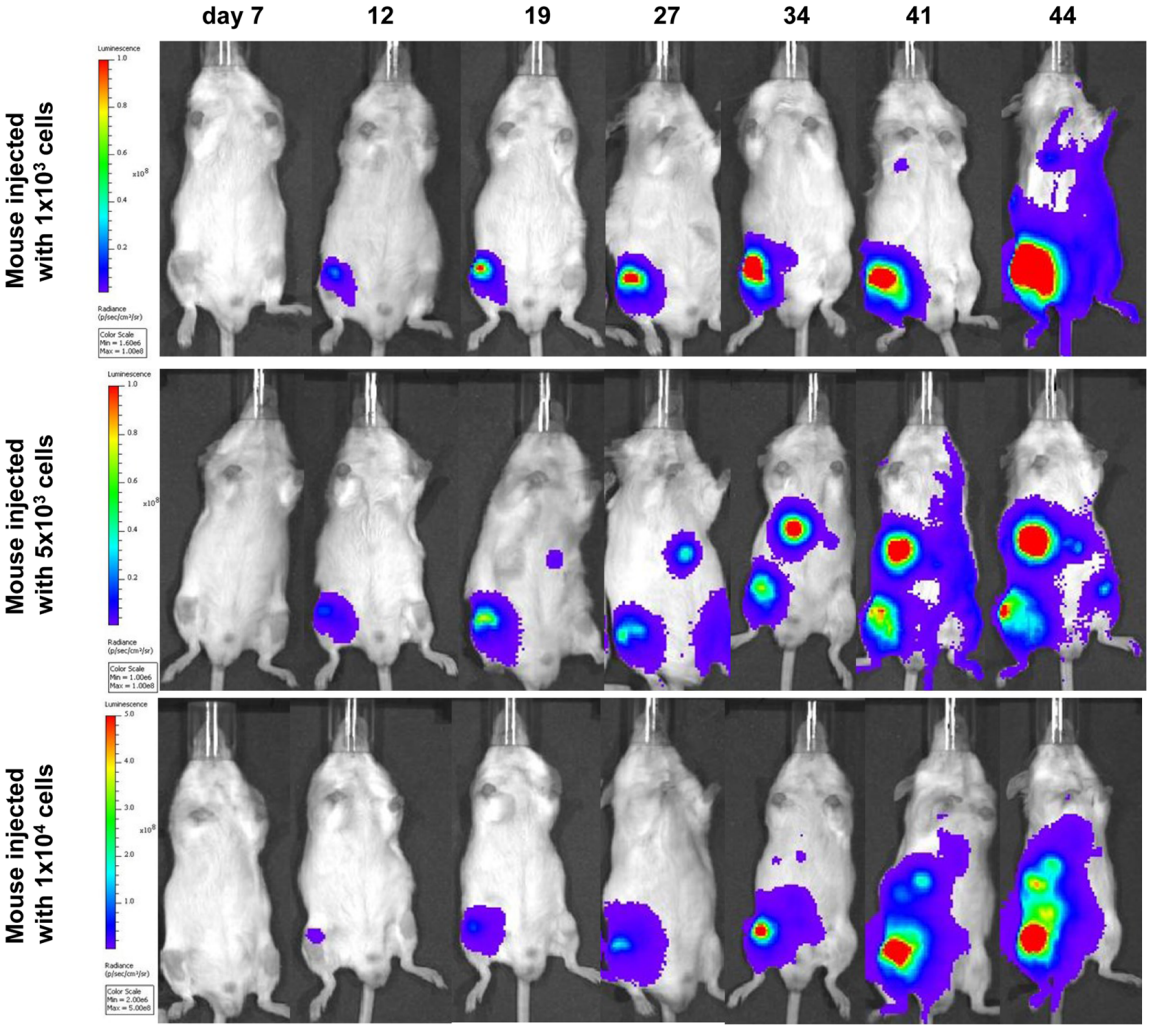
Bioluminescent imaging of of Rag2^−/−^ γc^−/−^ mice intrafemorally transplanted with low numbers of transduced TC-71 cells. Weekly bioluminescent imaging of 3 Rag2^−/−^ γc^−/−^ mice transplanted with 1×10^3^ (top row), 5×10^3^ (middle row) or 1×10^4^ (bottom row) transduced TC-71 cells (EGFP sorted 1 week prior to injection). Injected cells were suspended in a volume of 10 µl. The images obtained on day 7 did show a low signal over the right femur for all of the mice, but due to the chosen radiance settings to enable comparison with subsequent iamges, they do not appear on this panel. On some images of mice who were not imaged individually towards the end of the experiment (i.e. middle row day 27 and 41), the reflection of a signal emitted from a neighbouring mouse is unfortunately projected onto the left femur (day 27) or left side (day 41) of that mouse. All mice developed distant disease, either in lungs (top row), liver/abdomen (middle and bottom row) or contralateral leg (middle row).

All but one Rag2^−/−^ γc^−/−^ mice (12/13) injected with transduced TC-71 cells showed a detectable signal over the left femur as early as 4–7 days after i.f. injection, whereas tumours at the site of maximum signal emission over the femur became detectable by palpation 20 days after i.f. transplantation at the earliest (Figure 8, Figure 9 and Table 2). One mouse injected with 1×10^6^ TC-71 cells acquired an infection shortly after the i.f. injection and had to be culled on day 4 of experiment 1 after a positive bioluminescent signal over the femur was confirmed. The remaining mice lived for 24–62 days after i.f. injection. The mice injected with higher number of tumour cells (1–5×10^5^) had a median time of 31 days until reaching protocol limits, whereas those mice injected with lower number of cells (1×10^3^–1×10^4^) had a median time of 44 days before being culled.

**Table 2:** Summary of TC-71 bioluminescent signal detection in live mice and post-mortem (after dissection).

Organ	In live mice	Post mortem
femur	12/13	4/4
lungs	4/11	4/5
kidneys	1/11	1/5
Liver/abdomen	4/11	1/5
distant bone	3/11	2/5

One mouse with an IVIS-positive femoral tumour was culled on day 4 and could not be assessed for metastatic spread. Post mortem bioluminescence imaging of dissected organs was performed on 5 animals. The involved leg of one of these had been removed before imaging.

**Figure 10:**
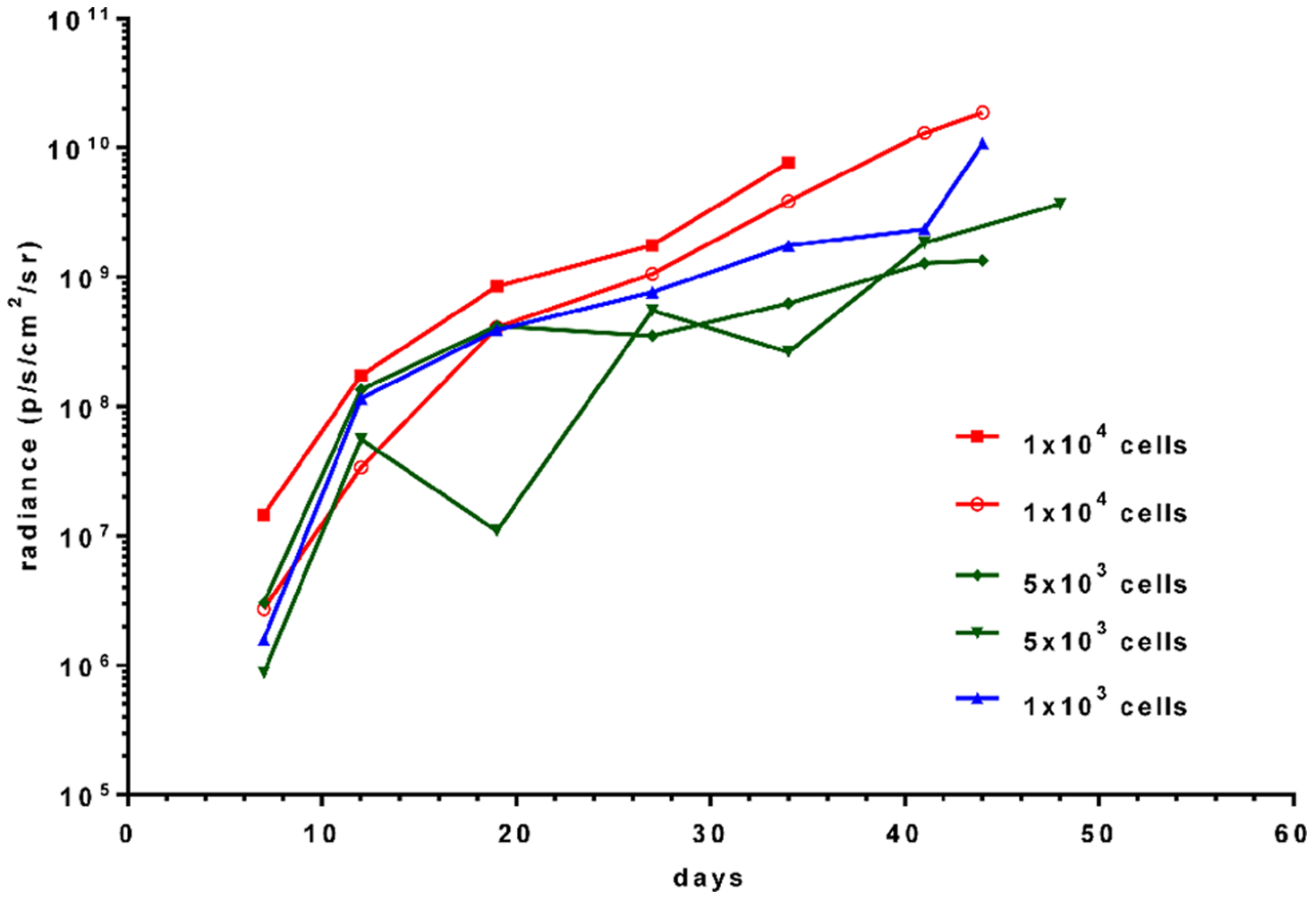
Timecourse of bioluminescent signal in mice injected with low numbers of transduced TC-71 cells. Depicted is the development of signal intensity for 5 animals injected with either 1×10^3^, 5×10^3^ or 1×10^4^ cells in a volume of 10 µl. The experiment was performed with EGFP sorted cells one week prior to injection.

**Figure 11:**
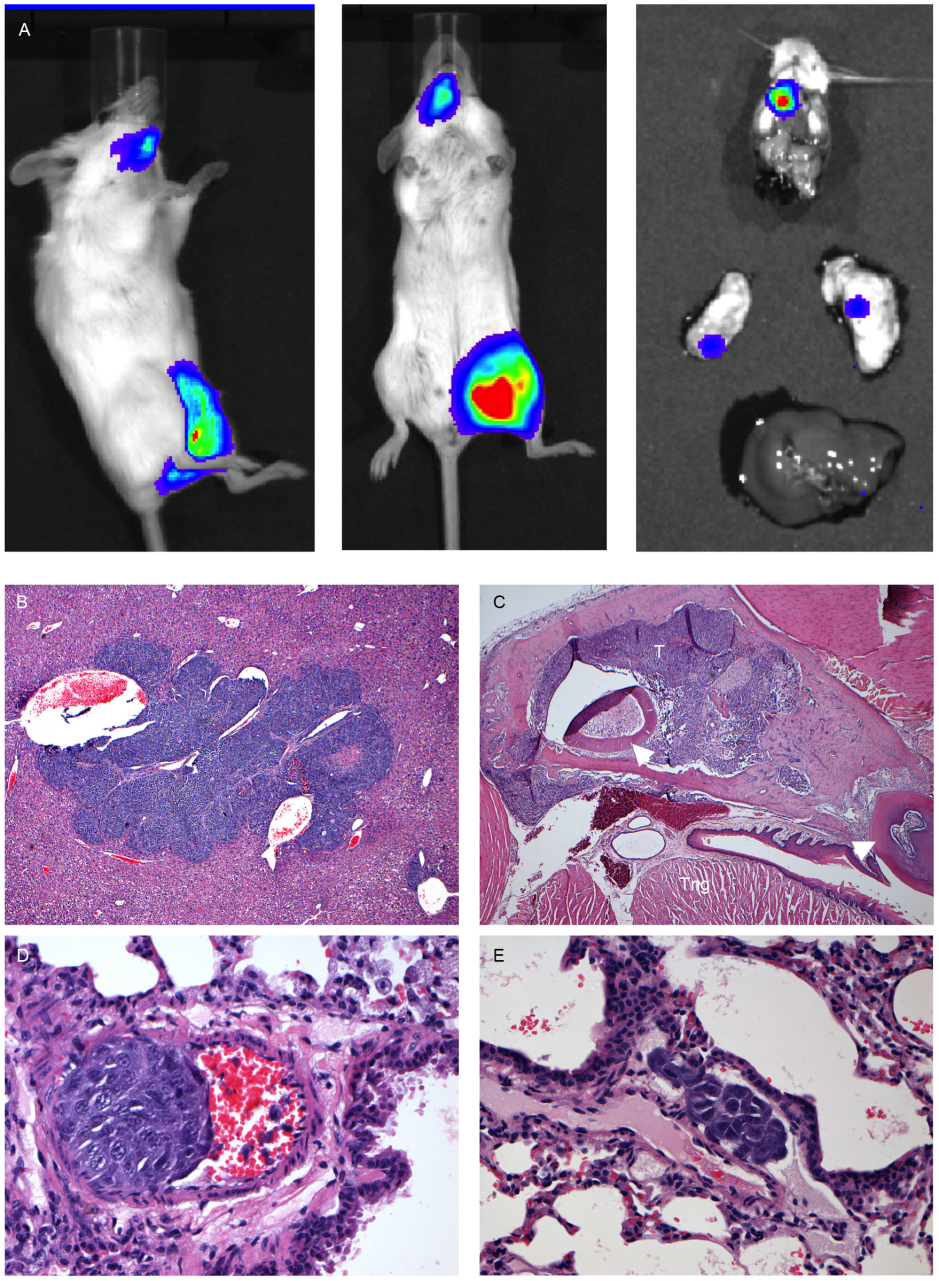
Examples of bioluminescent imaging of tumour spread to bone, post mortem imaging of organs, and.the histology of tumour spread. **A,** Example of a mouse transplanted with 1×10^5^ 25% EGFP positive TC-71 cells with metastatic spread to the jaw, and post mortem IVIS imaging of individual organs (dissected head, lungs and liver) demonstrating the signal from the jaw and additional weak signals from both lungs, all of which were confirmed by histology. **B,** TC-71 tumour deposit contained completely within liver parenchyma as an example of visceral tumour spread (H&E, original magnification 40×). **C,** TC-71 tumour deposit (T) within the mandible as an example of spread to distant osseus sites. Tng: tongue; arrowheads: teeth (H&E, original magnification 40×). **D,** Cluster of TC-71 tumour cells within a small arterial vessel adjacent to a bronchiole as an example of haematogenous spread (H&E, original magnification 400×). **E,** Cluster of TC-71 tumour cells within a lymphatic vessel adjacent to an arterial vessel and bronchiole as an example of intralymphatic spread (H&E, original magnification 400×).

For the mice injected with lower cell numbers (1×10^3^–1×10^4^) in experiment 3, the increase in radiance (p/s/cm^2^/sr) over the primary tumour sites with time is depicted in Figure 10. The signal intensity at day 7 varied for mice injected with equal number of tumour cells, with no clear correlation between cell number and signal intensity. In addition to the primary signal over the injected femur, signals reflecting tumour spread to lungs, bones or liver/abdomen could be detected from 3–4 weeks after i.f. transplantation. Disease spread to either lungs (n = 4), bone (n = 3), liver/abdomen (n = 4) or kidney (n = 1) was detected in 9 out of 11 mice by bioluminescent imaging in live mice (Figure 8, 9 and 11; Table 2). After dissection, the lungs of one additional animal (Figure 11), the head of another animal, and the liver/kidney of a further animal were found to emit a weak signal whereas previously no such signal could be detected over the respective fields of the anaesthetized whole mice. In all but one case examined, organs or tissues with a positive signal on *in vivo* imaging had tumour confirmed by macroscopic examination at autopsy or by histology. Tumour deposits presented as discrete nodules, 0.5–12 mm in diameter, within the organ parenchyma. In some lungs, tiny intravascular clusters of tumours cells, not related to any larger deposits, were seen indicating haematogenous or intralymphatic tumour spread (Figure 11). On dissection, 2 mice which were transplanted with 25% transduced TC-71 cells were found to have additional ovarian tumours not detected by bioluminescent imaging and likely to have arisen from non-transduced cells injected. In summary, bioluminescent imaging proved to be an excellent method to quantify the disease at the primary site and also to monitor distant tumour spread in a timely and non-invasive manner.

## Discussion

In this study we have developed preclinical orthotopic models of Ewing sarcoma and other primary and metastatic bone tumours by injecting cells directly into the femur of young NSG or Rag2^−/−^ γc^−/−^ mice. For Ewing sarcoma, our model is the first to combine an intraosseous orthotopic approach (i.e. injection *into* bone, in contrast to periosteal or intramuscular injections) with evaluation of different imaging approaches to monitor tumour growth and dissemination, including disease tracking by bioluminescent imaging. To our knowledge, there is only one published Ewing sarcoma model using bioluminescent imaging to date, in which transduced Ewing cells are injected into the periosteum/adjacent to a rib, giving rise to chest wall tumours and pulmonary metastases [30]. This model is more reflective of a soft tissue Ewing sarcoma or tumour growing into the bone from the periosteum than a truly intraosseous tumour. Other groups have examined in various ways the interactions of human Ewing sarcoma, osteosarcoma and prostatic carcinoma cells with murine bone in orthotopic models [19]; [20]; [31]–[35]. In the present study we extend this knowledge, showing histologically and radiologically that each tumour type interacts with the murine bone in a specific way resembling human clinical disease, validating our model as one in which the growth, spread and treatment of Ewing sarcoma can meaningfully be investigated.

Although other orthotopic approaches (e.g. intratibial injection) are available [18]; [19]; [20], our model offers several advantages. One advantage of an intrafemoral model is the good accessibility of the femur achieved by bending the knee and accessing it through the distal end. As with intratibial transplantation, intrafemoral injection is minimally invasive and non-traumatic, but the larger size of the femur can facilitate the transplantation procedure. The technique can be performed on animals from approximately 6 weeks of age. Secondly, the NSG and Rag2^−/−^ γc^−/−^ mice used are severely immunocompromised, allowing tumour induction in almost 100% of animals injected. Previous work has used intravenous injections to model the metastasis of Ewing sarcoma cell lines *in vivo* but bone metastasis is uncommon in this model and metastases hard to identify [16]. In the present orthotopic model, the observed pattern of spread of Ewing sarcoma cells was similar to the human cancer (primarily lung and bone), supporting the clinical relevance of the model. The histological findings are most consistent with a haematogenous or intralymphatic mode of tumour spread. However, although the appearance of distant tumour spread was significantly delayed in comparison to development of the primary tumour, the possibility that some tumour spread resulted from inadvertent innoculation of tumour cells into the circulation at the time of i.f. injection cannot entirely be excluded. Reassuringly, we only ever saw tumour involvement of the synovium or capsule of the knee joint in association with late-stage destruction of the distal femoral cortex induced by outgrowth of intrafemoral tumour, which makes significant spillage from the needle track back into the knee joint unlikely. We also initially observed ovarian metastases in two Rag2^−/−^ γc^−/−^ mice. As ovarian metastases are rather rare in the human disease, but have been observed in female animals from previous experiments [16], further experiments were conducted in male mice only, in order to better mirror the metastatic spread of Ewing sarcoma in humans.

To reduce the chance of tumour cell injection into the circulation, and also to not “overwhelm” the bone marrow cavity with large numbers of tumour cells, during development of the model we reduced the volumes injected by 66% (from 30 µl to 10 µl) and the number of tumour cells 100–1000 fold (down to 1000 cells). The resulting histological findings at post-mortem and the bioluminescent signals over time were very similar to earlier experiments using more cells in a higher volume, albeit with a slightly longer time for the animals to reach protocol limits (median 31 days for animals injected with 1–5 × 10^5^ cells vs. median of 44 days for animals injected with 1×10^3^–1×10^4^ cells). For other published Ewing sarcoma models, cell numbers vary from 2×10^5^ cells in 20 µl injected into the tibia [20], to 5×10^5^ cells in 20 µl injected around the periosteum of a rib [30] or to 1–2×10^6^ cells injected into the gastrocnemius muscle [36], [37] or the tibia [19]. Thus, we have here used the lowest number of cells for an orthotopic intraosseous injection reported in the literature so far, making this model the least likely to cause any non-physiological local side effects.

As the ability to image growing tumours greatly enhances the preclinical utility of *in vivo* tumour models, we assessed the usefulness of FDG-PET, CT, MRI and bioluminescent imaging in our orthotopic model. Previous work has used FDG- and [^18^F]-PET to examine metastatic tumour sites in mice transplanted intravenously with Ewing sarcoma cell lines [38]. However, due to the evaluation of summation plots as opposed to individual planes in this study, the tumours in this intrafemoral orthotopic model showed a relatively low signal intensity compared to host organs. Thus, FDG-PET was of limited value in the assessment of the tumours and detection of possible metastases. In contrast, MR imaging proved superior for the monitoring and measurement of the extra-osseous soft tissue components of the tumours induced. Additionally, in half of the mice that underwent an abdominal scan, MRI was able to detect metastases to the liver or peritoneal cavity. As expected, CT imaging provided excellent results for the visualisation of tumour-associated bone alterations, showing both the extent and distribution of reactive and “malignant” bone formation and the degree of bone destruction/resorption in the region of the implanted tumour. CT and MR imaging provided complementary information and such a combined modality approach is important for imaging bone tumours *in vivo*. Bioluminescent imaging was the superior method regarding practicability and performing imaging in a timely fashion for monitoring the development of primary and metastatic disease to different organs such as lungs, liver and bone, despite lacking the anatomical detail of CT or MRI imaging. However, tumour deposits arising from co-transplanted non-transduced tumour cells can lead to disease which is subsequently missed on bioluminescent imaging. Therefore, it is preferable to purify transduced cells before transplantation to minimise false negative results using vectors with selectable markers, such as EGFP. An even better consistency between signal and injected cell numbers and thus a better monitoring of disease burden over time might be obtained by selecting a monoclonal cell population for transplantation. Christoph et al. have recently compared monoclonal and polyclonal luciferase transduced cell populations *in vitro* and *in vivo* [39]. They have shown that for monoclonal populations of firefly luciferase transduced leukaemia cell lines, the luciferase activity was stable and light emission correlated strongly with cell numbers, leading to a consistent disease burden between animals.

By combining CT imaging with histological examination we have demonstrated that each of the cell lines used interacted with the bone microenvironment, altering bone structure, in characteristic ways. This finding confirms that our transplantation procedure preserves the mouse bone microenvironment in such a way that clinically relevant and tumour type-specific tumour-bone interactions may occur. Both Ewing sarcoma cell lines, particularly VH-64, induced florid reactive new bone formation consistent with pronounced periosteal reactions. In VH-64 transplanted mice, this was detected radiologically as spiculated new bone radiating perpendicularly from the cortex in a “sunburst” appearance. Although clinical Ewing sarcomas often produce a laminated “onion-skin” pattern of new bone, a “sunburst” pattern is seen in approximately 25% of cases [40]. The predominance of this pattern in the present model may reflect the relatively rapid tumour growth in the model compared to that in patients. While reactive new bone and cartilage formation may be seen in response to fracture, we do not believe that this is the predominant pathological process in our model as the degree of new bone formation was not correlated with the degree of bone destruction and fractures were seen in PC3M transplanted mice, which showed little new bone formation.

Injection of the murine femurs with cells of a human osteosarcoma cell line (SaOS-2) induced some reactive trabecular bone and cortical thickening, but most notable was the intra-tumoural deposition of osteoid, within which formed mineralised “malignant” bone. It can be concluded that this cell line is able to mature within the *in vivo* environment to form osteoid and bone in a manner similar to that seen in patients. The human prostate cancer cells (PC3M) caused pronounced osteolytic lesions within the murine femurs, leading to pathological fractures in some cases. This is in contrast to most bone metastases in advanced stage prostate carcinoma, which more commonly show osteoblastic/osteosclerotic characteristics. However, we have used this cell line as a model of osteolytic bone metastasis rather than metastatic prostatic carcinoma *per se*, and our findings are consistent with both the derivation of this cell line from a clinically osteolytic metastasis [24] and the generation of osteolytic bone lesions in other PC-3 xenograft models [18]. The different responses of the host bone to the different tumour types is consistent with a model in which specific reciprocal interactions between tumour cells and cells of the bone microenvironment cooperate to disturb normal bone homeostasis resulting in uncoupled bone remodelling. Experimental evidence suggests that these characteristic bone changes may support the growth of tumour in the metastatic environment [41] and they may also result in direct or indirect clinical morbidity. Understanding these interactions in models such as that described here is thus essential for the development of future therapies aimed at tumours within bone.

In conclusion, we have established an orthotopic human-mouse xenograft model that mirrors remodelling of normal bone by primary and secondary bone tumours and which will be of value for future studies analysing the interaction between malignant cells and the bone microenvironment. The model for Ewing sarcoma is the first reported combining an intraosseous orthotopic injection approach (using minimal numbers of injected cells) with bioluminescent imaging. The model could be further improved by selecting a single clone of transduced cells to achieve more consistent signal emission for different numbers of injected cells, or by transplanting primary patient material. In combination with MRI and bioluminescent imaging, our model has the potential to serve as a valuable tool for preclinical studies to assess the efficacy of newly developed pharmaceuticals for the treatment of both primary bone tumours and secondary metastases. Bioluminescent imaging allows for efficacious monitoring of disease progression and spread *in vivo*, which is a prerequisite for testing of anti-metastatic approaches. In summary, our models for a variety of bone cancers, including the first with the potential for *in vivo* tracking of Ewing sarcoma, will widely benefit bone cancer research and promote the translation of novel therapeutic approaches into clinical practice.

## Supporting Information

Figure S1 **Histology and CT imaging of a control mouse.** **A,** Representative CT image (near sagittal section through the right knee and femur) 42 days after injection of a control mouse injected with medium alone, showing normal appearances. **B,** Representative Maximal Intensity Projection CT image 85 days after injection depicting both lower extremities of a control mouse injected with medium alone, showing normal apperances. **C,** Representative histological section of right femur from a control mouse injected with medium alone, showing normal bone and skeletal muscle (H&E, original magnification 40×).
(TIF)

Figure S2 **MRI imaging of control mouse and mouse injected with SaOS-2 cells.** **A,** MRI scan on day 85, showing the legs of a control mouse injected with medium alone. **B,** MRI scan on day 85, showing a tumour in the right leg of a mouse injected with SaOS-2 cells (arrow).
(TIF)
