# Supplementary figures and images for: Development of a Preclinical Orthotopic Xenograft Model of Ewing Sarcoma and Other Human Malignant Bone Disease Using Advanced In Vivo Imaging
Source: PLoS One. 2014 Jan 7;9(1):e85128. doi: 10.1371/journal.pone.0085128 (PMC3883696; doi:10.1371/journal.pone.0085128)

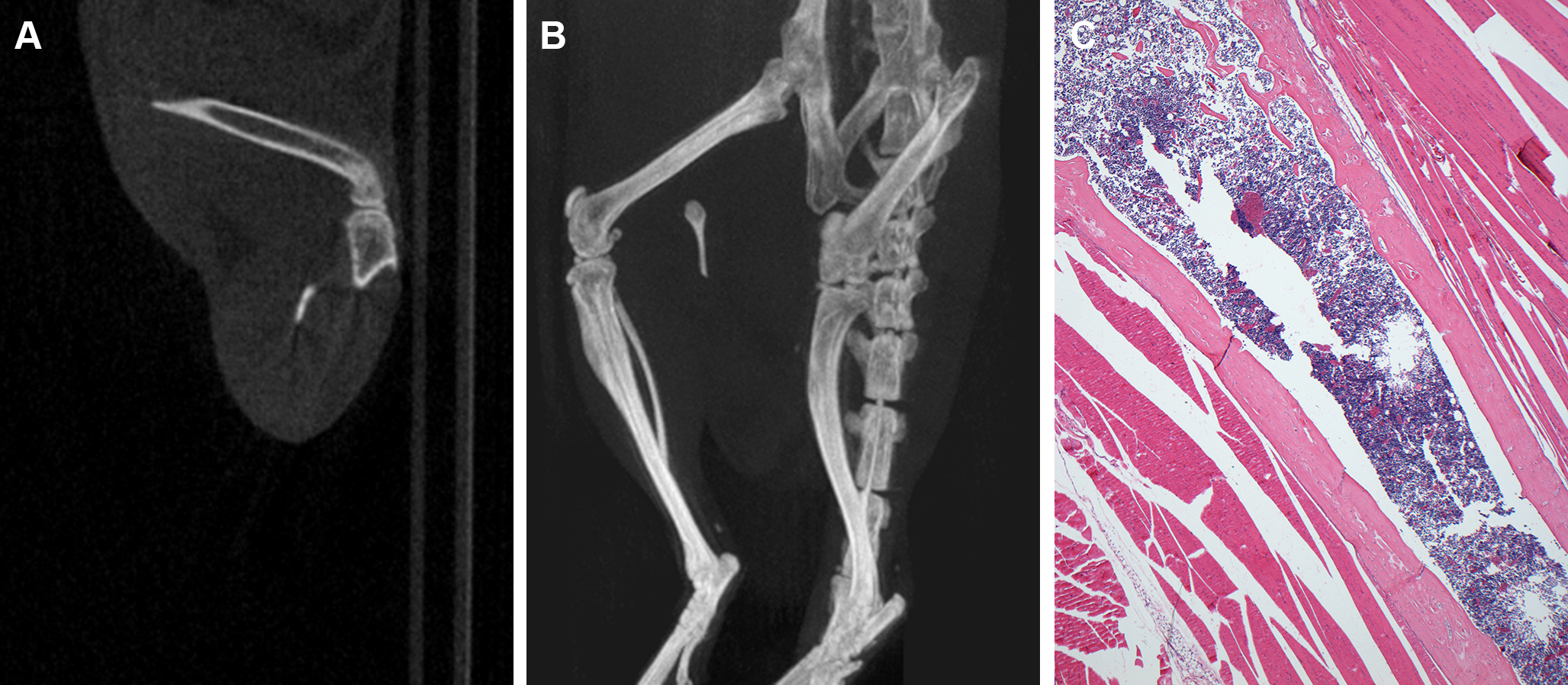

Supplement: Figure S1 — Histology and CT imaging of a control mouse. A, Representative CT image (near sagittal section through the right knee and femur) 42 days after injection of a control mouse injected with medium alone, showing normal appearances. B, Representative Maximal Intensity Projection CT image 85 days after injection depicting both lower extremities of a control mouse injected with medium alone, showing normal apperances. C, Representative histological section of right femur from a control mouse injected with medium alone, showing normal bone and skeletal muscle (H&E, original magnification 40×). (TIF) [file pone.0085128.s001.tif]

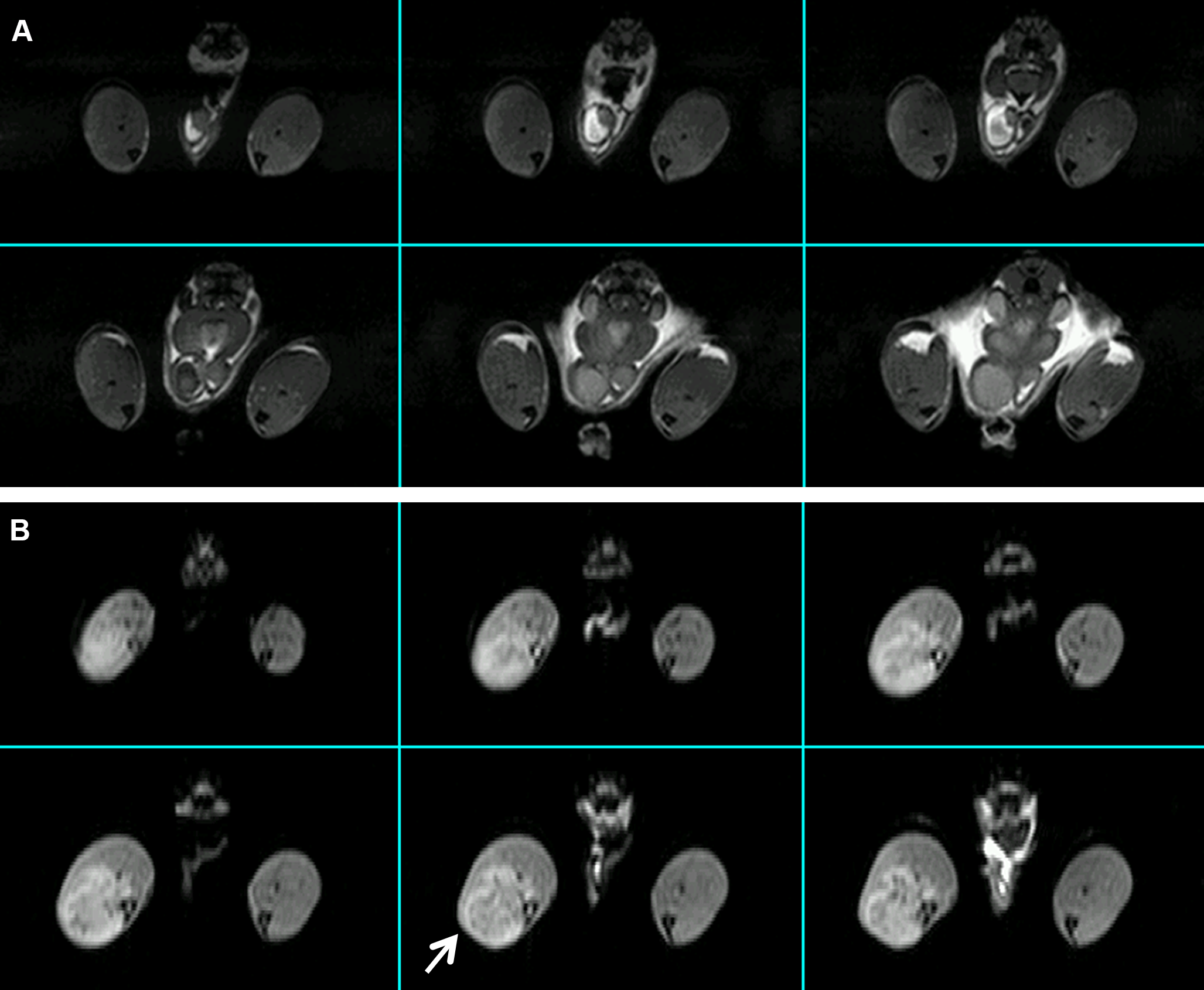

Supplement: Figure S2 — MRI imaging of control mouse and mouse injected with SaOS-2 cells. A, MRI scan on day 85, showing the legs of a control mouse injected with medium alone. B, MRI scan on day 85, showing a tumour in the right leg of a mouse injected with SaOS-2 cells (arrow). (TIF) [file pone.0085128.s002.tif]
